# Treatment modalities for patients with gambling disorder

**DOI:** 10.1186/s12991-017-0146-2

**Published:** 2017-04-28

**Authors:** Sam-Wook Choi, Young-Chul Shin, Dai-Jin Kim, Jung-Seok Choi, Seohee Kim, Seung-Hyun Kim, HyunChul Youn

**Affiliations:** 1 Korea Institute on Behavioral Addictions, True Mind Clinic, F7, KR tower, 1 141, Teheran-ro, Gangnam-gu, Seoul, 06132 South Korea; 2 Healthcare & Information Research Institute, Namseoul University, 91 Daehak-ro, Seonghwan-eup, Seobuk-gu, Cheonan-Si, Chungcheongnam-do 31021 South Korea; 3 Department of Psychiatry, Kangbuk Samsung Hospital, Sungkyunkwan University School of Medicine, 29 Saemunan-ro, Jongno-gu, Seoul, 03181 South Korea; 4 Department of Psychiatry, Seoul St Mary’s Hospital, College of Medicine, The Catholic University of Korea, 222 Banpodae-ro, Seocho-gu, Seoul, 06591 South Korea; 5 Department of Psychiatry, SMG-SNU Bora-mae Medical Center, 20 Boramae-ro 5-gil, Dongjak-gu, Seoul, 07061 South Korea; 6 Department of Psychiatry, Korea University Guro Hospital, Korea University College of Medicine, 148 Gurodong-ro, Guro-gu, Seoul, 08308 South Korea

**Keywords:** Gambling disorder, Pharmacotherapy, Psychosocial treatment, Opioid receptor antagonist, Cognitive behavioral therapy, Gamblers anonymous

## Abstract

**Background:**

Gambling disorder (GD) is defined as persistent and recurrent problematic gambling behavior leading to clinically significant impairment or distress. The prevalence of GD has been shown to be 1.2–7.1% in the general population. GD can severely impact on personal and vocational wellbeing as well as lead to financial problems, and has been known to be difficult to treat. This review describes the available pharmacotherapy/psychosocial treatments for GD patients, and summarizes data on the effectiveness of these GD treatments.

**Methods:**

This review refers to newly as well as previously published studies and guidelines.

**Results:**

The description of pharmacotherapy mainly focuses on opioid receptor antagonists, selective serotonin reuptake inhibitors, and mood stabilizers. Psychosocial treatments/strategies mainly include cognitive behavioral therapy, motivational interviewing, and Gamblers Anonymous. We also introduce relatively novel treatment modalities.

**Conclusions:**

This review can help clinicians to decide treatment plans for their GD patients. In addition, it can be used as a reference for designing future research.

## Background

Gambling disorder (GD) is defined as “persistent and recurrent problematic gambling behavior leading to clinically significant impairment or distress.” [1] Unlike the long history of substance addiction, GD has rarely been studied as a disease [2]. The American Psychiatric Association regarded “pathological gambling” as an impulse control disorder in the fourth edition of the *Diagnostic and Statistical Manual of Mental Disorders* (DSM-IV) [3]. In DSM-5, the most recent version, pathological gambling was re-categorized into the group of substance-related and addictive disorders and renamed GD [1]. This diagnostic change reflects longstanding conceptualizations of GD as an addiction [4].

Generally, the prevalence of GD has been shown to be 1.2–7.1% in the general population [5]. GD can severely impact on personal and vocational wellbeing as well as lead to financial problems [6, 7]. In addition, GD presents many psychiatric comorbidities such as depressive disorder, antisocial personality disorder, anxiety disorder, attention deficit hyperactivity disorder, and substance-related disorders [8, 9]. GD also can be associated with death due to suicide [10]. Various marketing tactics that gambling industry use recently may promote gambling problems [11, 12]. In South Korea, GD has become an important social problem. A national survey of 20,000 individuals from the general population reported that 5.4% had gambling problems [13]. The social costs of gambling are estimated to be over 11 trillion South Korean Won (KRW) (about 10 billion USD) per year [13].

To address these problems and social costs, effective interventions for GD patients are necessary. However, GD is notoriously difficult to treat, as it is a chronic relapsing disorder with high treatment dropout rates [14]. Therefore, we believe that clinicians need to pay more attention to the treatment of GD. Treatment modalities for GD have generally been classified into two categories: pharmacotherapy and psychosocial treatments [15]. Pharmacotherapy includes not only anti-craving agents but also antidepressants and mood stabilizers. Group/individual cognitive behavioral therapy (CBT), motivational interviewing (MI), and Gamblers Anonymous (GA) are examples of psychosocial treatments [10]. In this review, we describe pharmacotherapy/psychosocial treatments currently available, and summarize data on the effectiveness of these GD treatments. In addition, we also introduce more recently tested treatment modalities. We refer to newly as well as previously published studies and guidelines, and conclude with recommendations for future research.

## Pharmacotherapy

No medication has received Food and Drug Administration approval as a treatment for GD to date [16]. However, pharmacotherapy can have positive effects on GD patients such as reduction of urge, treatment of comorbidities, and relapse in prevention [15]. Medications frequently used to treat GD are opioid receptor antagonists, selective serotonin reuptake inhibitors (SSRIs), and mood stabilizers.

## Opioid receptor antagonists

Opioid receptor antagonists have been used in the management of alcohol and drug dependent patients for several decades [17, 18]. Also in GD, these medications can diminish urges to engage in gambling and increase the periods of abstinence by modulating the effects of the arcuate nucleus opioid neurons on the ventral tegmental area and mesolimbic dopamine circuitry [19, 20]. A recent meta-analysis study showed that, compared to placebo, only opioid receptor antagonists demonstrated significant benefit in the pharmacological treatment of GD. However, this result provided only limited support due to its methodology [21]. Opioid receptor antagonists include naltrexone and nalmefene. In 2001, the first double-blind placebo-controlled study on the efficacy of naltrexone in GD was published [22]. This study involved an 18-week trial and showed that naltrexone is effective in reducing the intensity of urges to gamble, gambling thoughts, and gambling behavior. The efficacy of naltrexone was especially high in individuals with higher intensity of gambling urges. In this study, the mean daily dose of naltrexone was 188 mg, and many individuals reported side effects such as nausea, dry mouth, and vivid dreams. Grant et al. replicated these findings in a larger study [23]. They suggested that a dose of 50 mg of naltrexone was sufficient and associated with fewer side effects. In addition, one study reported that the positive effect of naltrexone may persist after discontinuation [24]. Naltrexone has also been shown to be effective in GD patients with Parkinson’s disease already treated with dopamine agonists [25]. On the other hand, Kobanen et al. tried to verify the effect of as-needed naltrexone through a randomized, double-blind, placebo-controlled trial [26]. They instructed participants to take one capsule always in an as-needed manner when planning to gamble or when experiencing a strong urge to gamble (preferably 30–60 min before gambling), but the rates of response did not differ between groups. Two large double-blind placebo-controlled studies reported that nalmefene demonstrated superior efficacy than placebo in treatment outcomes for GD [20, 27]. These studies suggested that 25, 40, and 50 mg of nalmefene were effective but higher doses increased side effects such as nausea.

## Selective serotonin reuptake inhibitors (SSRIs)

SSRIs were one of the first medication types tested for treating GD. Several studies reported that fluvoxamine was effective in the treatment of GD [28–30]. The treatment duration in these studies was 8 or 12 weeks, and the maximum doses of fluvoxamine were 200 or 250 mg. However, another study on fluvoxamine treatment in GD patients did not reach statistical significance [31]. In addition, Dannon et al. conducted a 6-month follow-up study for fluvoxamine responders who discontinued their medication [24]. They reported that three of six fluvoxamine responders relapsed. On the other hand, paroxetine was reported to be superior to placebo in one trial, but not in another. Kim et al. conducted a double-blind placebo-controlled trial, and showed the efficacy of paroxetine in the treatment of GD [32]. The mean doses of paroxetine were 51.7 mg, and adverse events were minimal. Nevertheless, another double-blind placebo-controlled trial reported that the paroxetine group showed larger improvements than the placebo group, but not at a significant level [33]. Furthermore, one open-label trial suggested that escitalopram was effective in the treatment of GD [34]. Another study also found a significant improvement in individuals with GD and co-occurring anxiety disorders [35]. However, further double-blind, placebo-controlled studies should be performed to support this result. In the case of sertraline, one double-blind placebo-controlled study was conducted to verify its efficacy, but the authors failed to draw significant results [36]. There is a controversy about whether SSRIs can reduce urges to gamble, but they can treat the anxiety and depressive symptoms of GD patients. In turn, this may reduce gambling behavior in patients who gamble to avoid anxiety and depression [15]. Additional double-blind placebo-controlled studies involving larger samples are needed to verify the efficacy of SSRIs more clearly. In addition, there was a tendency to require relatively high doses and long treatment duration for effective SSRI treatment of GD. Thus, future studies also need to focus on identifying optimal doses and treatment duration of SSRIs.

## Mood stabilizers

Mood stabilizers have also been used to treat GD. Hollander et al. conducted a double-blind placebo-controlled trial enrolling individuals with GD and bipolar-spectrum disorders to investigate the efficacy of lithium [37]. The authors found that the lithium group showed significantly less severe gambling urges and behavior than did the placebo group. On the other hand, one single-blind study compared lithium and valproate treatments in GD patients [38]. Both the lithium and valproate groups showed a significant improvement with similar efficacy, but the small sample size, single-blind design, and lack of placebo control were limitations of this study. Black et al. tested carbamazepine in the treatment of GD and reported a significant improvement [39]. However, this study provided limited evidence because of its open-label setting, small sample size, and lack of placebo control. One double-blind placebo-controlled trial showed that topiramate reduced impulsivity in GD patients but was not superior to placebo in reducing gambling urges and behavior [40]. On the other hand, a recent double-blind placebo-controlled study reported that topiramate proved to be superior to placebo in reducing gambling craving, time and money spent gambling, cognitive distortions related to gambling, and social difficulties [41]. However, this study used a brief cognitive intervention as well as topiramate or placebo; therefore, the results could be due to a synergistic intervention. The efficacy of mood stabilizers in GD has been investigated mainly in specific settings such as comorbidity with bipolar disorder; thus, more studies are needed to verify their efficacy.

## Other medications

Besides the abovementioned pharmacotherapies, various medications have been investigated in the treatment of GD patients. Bupropion has been used to treat various addictive disorders [42, 43]. Dannon et al. conducted a blind-rater study comparing GD subjects taking naltrexone and bupropion, and reported that the efficacy of bupropion was similar to that of naltrexone [44]. However, the first and only double-blind placebo-controlled trial examining bupropion showed that it was not superior to placebo in the treatment of GD [45]. Olanzapine has been evaluated in two double-blind, placebo-controlled trials, but both studies failed to show differences between olanzapine and placebo in the treatment of GD [46, 47]. In addition, Grant et al. reported the efficacy of as-needed ecopipam on GD patients in single-blind setting. However, more controlled studies are required to support this result [48]. On the other hand, one study reported that haloperidol actually promoted gambling-related thoughts and behaviors [49]. Zack and Poulos tested modafinil with GD patients in a double-blind, placebo-controlled setting [50]. They reported that modafinil decreased motivation to gamble and risky decision making, and improved inhibitory control in high-impulsivity participants; however, it had opposite effects on low-impulsivity participants. Another analysis showed that modafinil might deter pathological gamblers from chasing losses but also encourage them to continue betting rather than quit while they are ahead [51]. N-acetyl cysteine (NAC) was found to be effective in the treatment of GD patients in one open-label trial with a double-blind discontinuation period [52]. However, there were some limitations to this study such as small sample size, short duration of treatment, and the fact that only responders were randomized into the double-blind portion of the study. One double-blind, placebo-controlled trial tested NAC in individuals with co-occurring nicotine dependence and GD who were receiving imaginal desensitization therapy for GD and found that NAC presented additional benefits at 3-month follow-up [53]. Amantadine was shown to decrease GD symptoms among GD individuals with Parkinson’s disease in a double-blind, placebo-controlled trial [54]. A recent study also reported that GD participants with Parkinson’s disease showed a decrease in risky choices and an increase in non-risky choices during amantadine treatment [55]. On the other hand, another study found that amantadine use could be associated with GD in Parkinson’s disease patients [56]. One open-label study tested memantine in GD patients and reported that memantine treatment was associated with diminished gambling and improved cognitive flexibility [57]. However, this study provided limited evidence because of small sample size, short-term follow-up duration, and open-label setting. In two open-label trials, acamprosate has also been tested for GD patients. However, the result of each trial is conflicting [58, 59]. The efficacy of most of these medications to treat GD has not yet been definitely confirmed. Some of them have proved to be effective in only specific situations. In addition, a few of them were even found to exacerbate the symptoms of GD. Therefore, clinicians should be careful when prescribing these medications to GD patients.

## Psychosocial treatment

### Cognitive behavioral therapy (CBT)

CBT combines various aspects of both cognitive and behavioral techniques. Cognitive approaches focus on erroneous beliefs and biased information processing, while behavioral techniques are based on the assumption that problems are learnt maladaptive behavior. CBT has been the main psychological therapy for GD [60]. The targets of CBT for GD are correcting the cognitive distortion, decision making/reward processing, and physical/psychological responses that are associated with gambling [61]. CBT for GD includes cognitive correction, problem-solving training, social skills training, and relapse prevention [62].

Various studies have reported the efficacy of CBT in GD patients [63–66]. The content of CBT differs across studies in the extent of cognitive versus behavioral emphasis, but all include both elements to some degree [67]. In addition, some studies have shown that both individual and group-delivered CBT present similar efficacy in treating gambling behavior and in relapse prevention [68, 69]. Two meta-analyses have also confirmed the efficacy of CBT in GD patients [60, 70]. Recently, the efficacy of CBT has also been studied in various countries such as China and South Africa [71, 72]. Furthermore, the study protocol for a pragmatic randomized controlled trial evaluating the effectiveness in treating GD of CBT, behavior therapy, and MI against a non-directive supportive therapy control has been published [73]. Future research including the results of this study will provide more verified evidence for the efficacy of CBT.

### Motivational interviewing (MI)

MI was developed as a way to help individuals work through ambivalence and commit to change [74]. MI therapies refer to any treatment that is based predominantly on a MI approach [60]. MI explores the patients’ own arguments for change in an empathic and supportive manner. The interviewers help patients to express their desire, ability, reasons, and need for change, and respond with reflective listening [75]. Verbalized intention results in an increased probability of behavior change, particularly when it is combined with a specific plan for implementation [76]. Therefore, MI involves two distinct phases: the first focuses on increasing motivation for change, and the second on consolidating commitment [75]. MI is often brief and can be delivered as a freestanding treatment or as a motivational prelude to other treatments. It has also been commonly combined with other intervention components. A motivational enhancement therapy, which combines MI with a standardized assessment of problematic behavior and personal feedback on results, is the most widely used combined intervention [60].

Several studies have investigated MI to treat GD patients and showed its efficacy. Different durations of treatment were used, but tended to be brief. Carlbring et al. conducted four 50-min sessions [63], and Toneatto and Gunaratne conducted six weekly sessions [77]. On the other hand, many studies adopted only one session of MI [66, 78–80]. The efficacy of MI in GD patients was also confirmed by meta-analytic data [60, 81]. Whether treatment effects are maintained over time remains unclear [81], and further studies on long-term effects are thus needed.

### Gamblers anonymous (GA)

GA is a mutual aid fellowship based on 12-step principles founded in the 1950s [82]. Mutual aid is a way to bring individuals together to address a shared problem [83]. Alcoholics Anonymous, Narcotics Anonymous, and groups for people with cancer are examples of mutual aid [83]. In terms of the mutual aid principle, Kelly et al. reported that the group processes in Alcoholics Anonymous could augment self-efficacy, coping skills, and motivation by helping individuals build supportive and pro-social networks [84]. GA involves a similar principle but, unlike other mutual aid groups, it also focuses on financial and legal problems caused by gambling [85, 86]. GA has long been an accessible option for persons seeking help for a gambling problem in most North American cities [83, 87]. In South Korea, GA was founded in 1984, and currently has more than 30 branches [15].

Some randomized controlled trials have examined the effectiveness of referral to GA, but these studies have dealt with GA as an adjunct treatment or as a controlled condition [65, 88–90]. Therefore, further studies are needed to explore the independent efficacy of GA, for instance, adopting a waitlist control group.

### Other treatment approaches

In addition to the abovementioned pharmacotherapy and psychosocial treatments, other treatment modalities have been proposed for GD patients. Hedman et al. compared internet-based CBT to conventional CBT, and reported that internet-based CBT involved lower costs and showed equivalent efficacy [91]. For treating GD patients, Calbring and Smit suggested CBT with minimal therapist contact via e-mail and a weekly telephone call [92], while Hodgins and Makarchuk developed online versions of the self-change tools [93]. Recently, many studies on the treatment of GD have adopted internet-based approaches [94–96]. Most of these studies translate conventional treatment methods to online-based therapy; however, we believe that future studies can develop more internet-specific therapies. Smartphones are internet-based and have a wide range of functions and high availability [97, 98]. Recently, smartphones have been increasingly used to treat addictive disorders such as alcohol and substance abuse [99–101], and we believe that they can also be considered as a tool for treating GD patients.

Additionally, Linardatou et al. adopted an eight-week stress management program for GD patients participating in GA [88]. The stress management program consisted of education on diet/exercise, stress coping methods, relaxation breathing, and progressive muscle relaxation; and it was found to be effective in decreasing stress, depression, and anxiety, and improving life-satisfaction and daily routine. Zack et al. tried high-frequency repeated transcranial magnetic stimulation (rTMS) and continuous theta burst stimulation in GD patients, and reported that these can reduce gambling reinforcement [102]. In addition, Gay et al. and Rosenberg et al. also tested the effect of rTMS and deep TMS on GD patients, respectively [103, 104]. However, there are few studies on these treatments and more studies are thus needed for their wider clinical application.

Recent studies are being conducted on the effectiveness of combined pharmacotherapy and CBT for the treatment of GD [105]. One study of CBT versus escitalopram combined with CBT reported that escitalopram did not appear to enhance the CBT treatment outcome [106]. Choi et al. conducted a retrospective chart review to compare treatment duration according to pharmacotherapy and group CBT [107]. They reported that combined group CBT and any type of medication showed significantly longer treatment maintenance duration than pharmacotherapy or group CBT alone. Especially, combined antidepressant pharmacotherapy and group CBT was the most effective intervention in maintaining the treatment. Until now, studies on the efficacy of these combination therapies have been insufficient, but we believe that clinicians can try these treatment options depending on the situation.

## Conclusions

Despite significant advances in research, our understanding of treatment strategies for GD remains relatively poorer than for other major neuropsychiatric disorders. In this study, we described the most updated versions of pharmacological and psychosocial treatments for GD patients. Especially, we made an effort to include recently published meta-analyses and randomized controlled trials. We also introduced other novel treatment modalities. As GD is a heterogeneous disorder, clinicians need to choose the best treatment for their GD patients among various treatment modalities depending on the situation. Our study provides various treatment options and related evidence, including pharmacotherapy and CBT, and we therefore believe that our results can help clinicians to choose the best options. In addition, there are still not enough studies on GD treatment; thus, more studies—especially meta-analyses and randomized controlled trials—are needed. Abovementioned novel treatment options such as combined pharmacotherapy/CBT and therapies using internet/smartphones also need to be further examined and tested. We expect that future studies will suggest more verified and diverse treatment modalities for GD patients.

## Abbreviations

GD: gambling disorder
DSM: diagnostic and statistical manual of mental disorders
KRW: South Korean Won
CBT: cognitive behavioral therapy
MI: motivational interviewing
GA: gamblers anonymous
SSRIs: selective serotonin reuptake inhibitors
NAC: N-acetyl cysteine
TMS: transcranial magnetic stimulation
